# Editorial: Women in breast cancer, volume III: 2023

**DOI:** 10.3389/fonc.2024.1515282

**Published:** 2024-12-03

**Authors:** Francesca Bianchi, Anika Nagelkerke

**Affiliations:** ^1^ Department of Biomedical Health Sciences, University of Milan, Milan, Italy; ^2^ Laboratorio di Morfologia Umana Applicata, IRCCS Policlinico San Donato, Milan, Italy; ^3^ Department of Pharmaceutical Analysis, Groningen Research Institute of Pharmacy, University of Groningen, Groningen, Netherlands

**Keywords:** breast cancer, personalized medicine, quality of life, gender equality, prevention, leadership, fertility

## Introduction

Breast cancer is still the most common cancer among women worldwide, causing a heavy burden of disease, mortality and long-term survival. In this context, women’s participation in breast cancer research, especially in the leadership process, is important for increasing knowledge and developing health policy. However, gender gaps in leadership still exist. According to the 2023 OCSE report, women make up less than 30% of scientists worldwide, and their representation in senior positions and as decision-makers is even lower, especially in programs such as medical and experimental oncology. This under-representation not only hinders development of new ideas to solve complex diseases, such as breast cancer, but also impedes the progress needed to solve the specific health problems faced by women.

Negative factors such as research funding bias, organizational bias, and lack of continuing education risk hindering women’s advancement in scientific research. Furthermore, as a result of their significant contributions to research, female researchers should be recognized more for their contributions, leadership, and talent in relevant publications. The COVID-19 pandemic has further exacerbated inequality, negatively impacting female researchers who face greater supervisory responsibilities, thereby reducing research output. As we strive for a more social environment, it is even more important to provide platforms that support and promote the work of female scientists.

The Frontiers in Oncology’s Women in Breast Cancer series provides one such platform to share the diverse, valuable latest contributions of female researchers to breast cancer research. Volume III contains 21 articles covering a wide range of topics from molecular analysis and new diagnostics to clinical and patient care. These projects are all led by women and include a large number of female scientists, at various stages in their careers. Collectively, this series provides insight into the evolution of breast cancer research, providing a deeper understanding of early treatment and the biological, psychological, and treatment of the disease.

## Innovations in non-invasive diagnostics

Advances in breast cancer diagnostics are critical to improving early diagnosis and ensuring that patients receive the best possible treatment and personalized care. A key study in this volume investigates the ability of ultrasound-based radiography to estimate the expression of important markers such as oestrogen receptor (ER), progesterone receptor (PR), HER2, and Ki-67 in breast cancer patients (Xu et al.). This new approach aims to eliminate molecular testing, reduces the need for biopsies, and offers a more efficient, patient-friendly alternative to traditional diagnostics. These advances are particularly important for improving diagnostic accuracy and guiding treatment in different types of breast cancer.

This research is complemented by another study on breast calcification, which is often an early sign of malignancy. A new machine learning algorithm was developed to improve the segmentation and quantification of calcium in mammograms (Tong et al.). The algorithm is superior in identifying microcalcifications compared to existing models, improving the diagnostic process by helping radiologists distinguish malignant diseases. This AI-powered innovation holds great promise for improving early detection and reducing misdiagnosis, especially in difficult-to-find early-stage breast tissue.

## Personalized therapy and radiotherapy

Advances in decisive oncology continue to improve breast cancer treatment. Much of the research in this Research Topic focuses on personalized treatment strategies to improve outcomes based on the patient’s molecular profile. A key example is the study of the Lem-D protein in triple-negative breast cancer (TNBC) (Rose et al.). Because TNBC lacks hormone receptors that are frequently targeted by other types of breast cancer, treatments are often limited. The researchers in this volume describe the role of nuclear envelope proteins, such as Ankle2, TMPO, and Emerin in TNBC cell growth, providing compelling evidence that these proteins may serve as a new treatment plan. This opens the door to more effective, targeted interventions for one of the most serious and difficult-to-treat breast cancers.

Improving of personalized therapy also include traditional treatment such as radiotherapy. Comparative studies of conventional and hypofractionated radiation therapy using the Halcyon system highlight the importance of radiation therapy to stabilize the tumour with reduced side effects (Tai et al.). The hypofractionation approach, which delivers more radiation in fewer sessions, has been shown to be better minimizing side effects, even if not as effective in controlling tumours, as than conventional ones.

## Quality of life impact

Breast cancer, irrespective of its extent and treatment, has negative impacts on several aspects of women’s lives. This Research Topic includes several studies that highlight the importance of health-related quality of life (HR-QoL) and patient outcomes, particularly in light of the COVID-19 pandemic. One study examined how the COVID-19 vaccine impacted HR-QoL in patients with breast and gynaecological cancer (Forster et al.). Findings suggest that vaccination helps improve patients’ overall health, reduces stress, and improves their ability to participate in daily life. Given cancer patients’ vulnerability to infection, these results highlight the important role of vaccination in reducing further health risks and improving mental health during a global health crisis.

A common complication after breast cancer surgery that also affects patients’ quality of life is lymphedema. A study of clinical decongestant therapy (CDT) in women with postmastectomy lymphedema showed that the treatment resulted in significant improvements in physical health and pain intensity (Kavak and Ünver). Patients had increased mobility, reduced limb size, and improved health through exercise and education services. These findings emphasize the importance of incorporating rehabilitation into breast cancer treatment plans to ensure that holistic care continues beyond the postoperative period.

Fertility preservation is another important issue, especially for young women diagnosed with breast cancer. The reviews and assessments in this volume highlight the concerns related to pregnancy in young cancer patients, with over 50% of patients reporting serious concerns about becoming pregnant after treatment (Chen et al.). Factors such as education level, reproductive stress, and depression have been found to be important contributors to these stressors, while having a partner or having children has been shown to be protective. This study shows the need for counselling and support that not only affects the physical health of the patient, but also the patient’s mental and emotional health among young women with cancer. In addition, data on pregnancy-associated cancer (PABC) highlight the unique challenges in cancer management during pregnancy (Innocenti et al.). The case describes the success of a patient who underwent breast-sparing mastectomy and immediate breast reconstruction. This multifaceted exercise emphasizes the importance of clinical strategies that define maternal and foetal health to ensure that breast cancer care is appropriate for reproductive outcomes.

## Cancer prevention

The relationship between genes and breast cancer has been an important topic of research. The main report in this volume is about inflammatory breast cancer (IBC), a rare and serious disease (Qin et al.). Through genetic analysis, researchers have identified the most important mutations, such as PIK3CA, and the mechanisms that can affect the recurrence of the disease. These data demonstrate the benefits of genetic analysis in guiding treatment strategies and identifying therapeutic targets, particularly in complex, recurrent cases.

In addition, research on the immune system and their association with cancer risk provides new insights into the possibility of immunotherapy. A Mendelian randomization study showed how immunosuppressants can increase or decrease the risk of cancer, especially in ER-positive and ER-negative subtypes (Wang et al.). These findings provide the basis for further research on immunotherapy, which can provide more personalized and effective treatments for patients with the disease.

## Conclusion

The material collected in “Women with Breast Cancer Vol III: 2023” shows the depth of breast cancer research led and performed by women. From innovations in research and genetic information to the development of personalized treatment strategies and a focus on mental health, these studies are pushing the boundaries of our understanding and approach to breast cancer. In addition, this book shows the importance of addressing the specific problems of young patients, especially reproductive and health problems, while strengthening our understanding of the genetics and immune system involved in the disease.

As the work continues, the role of women in leading and improving breast cancer research cannot be overestimated. By providing a forum for female researchers, Frontiers in Oncology not only amplifies their voice, but also ensures that their contributions are recognized and included in the general discussion. The findings and research presented in this Research Topic provide important guidance to clinicians, researchers, and policy makers.

